# Metric Learning-Guided Semi-Supervised Path-Interaction Fault Diagnosis Method for Extremely Limited Labeled Samples under Variable Working Conditions

**DOI:** 10.3390/s23156951

**Published:** 2023-08-04

**Authors:** Zheng Yang, Fei Chen, Binbin Xu, Boquan Ma, Zege Qu, Xin Zhou

**Affiliations:** 1 School of Mechanical and Aerospace Engineering, Jilin University, Changchun 130025, China; yangzhengjlu@163.com; 2 Sino-German College of Intelligent Manufacturing, Shenzhen Technology University, Shenzhen 518118, China; xubinbin@sztu.edu.cn (B.X.); maboquan1025@163.com (B.M.); quzege@163.com (Z.Q.); zhouxinsztu@163.com (X.Z.)

**Keywords:** intelligent fault diagnosis, semi-supervised, metric learning, limited labeled sample, variable working condition

## Abstract

The lack of labeled data and variable working conditions brings challenges to the application of intelligent fault diagnosis. Given this, extracting labeled information and learning distribution-invariant representation provides a feasible and promising way. Enlightened by metric learning and semi-supervised architecture, a triplet-guided path-interaction ladder network (Tri-CLAN) is proposed based on the aspects of algorithm structure and feature space. An encoder–decoder structure with path interaction is built to utilize the unlabeled data with fewer parameters, and the network structure is simplified by CNN and an element additive combination activation function. Metric learning is introduced to the feature space of the established algorithm structure, which enables the mining of hard samples from extremely limited labeled data and the learning of working condition-independent representations. The generalization and applicability of Tri-CLAN are proved by experiments, and the contribution of the algorithm structure and the metric learning in the feature space are discussed.

## 1. Introduction

As one of the most important components of intelligent manufacturing equipment, the health status of rotating machinery may affect the overall operation status of the equipment. For instance, the faults of bearings are prone to reducing the processing quality of the workpiece, and even result in considerable economic losses and potential safety hazards. Each fault of rotating machinery will eventually be embodied in the external excitation caused by mechanical structure defects, which produces mechanical vibration signals which differ from the healthy state. Due to the exceptional performance in solving the nonlinear feature extraction for machine vibration data, deep learning has witnessed remarkable success in the field of rotating machinery fault diagnosis [1]. In practical engineering, however, the lack of labeled data and the variable working conditions will restrict the profound study of prognostic and health management for rotating machinery [2].

Recent advances in supervised learning methods have been widely employed to overcome the challenge of variable working conditions. Xing et al. [3] proposed a distribution-invariant deep belief network (DBN) to learn distribution-invariant features by a locally connected structure. Zhao et al. [4] converted the one-dimensional signal to a three-dimensional image and applied a multiscale inverted residual convolution neural network (CNN) to learn different representations of variable load bearings. The gate units of a long short-term memory (LSTM) network were also utilized to store and transfer the classification information [5], and thus the working condition information could be ignored while the health condition was emphasized. The attention mechanism [6,7] combined with transfer learning enabled the model to retain invariant fault representation related to the faults during the training process. Although the aforementioned methods show superiority and outstanding stability in dealing with the inconsistent distributions within data under variable working conditions, these implementations have limitations in practical industrial scenarios. Ordinarily, the training of a decision-making model is based on the assumption of abundant labeled data, but it is unrealistic to label massive data in industrial applications.

Researchers have mainly made great efforts to alleviate the problem of insufficient labeled data from these three aspects: the feature learning-based strategy, the algorithm structure-based strategy and the data augmentation-based strategy [8]. From the perspective of the feature learning-based strategy, feature transfer based on transfer learning attained satisfactory diagnostic results. He et al. [9] designed a deep multi-wavelet autoencoder to select high-quality auxiliary samples for parameter knowledge transfer. Li et al. [10] constructed a multi-layer CNN to extract transferable features from the limited labeled data of the source domain and reduced the discrepancy of the marginal and the conditional probability distribution for limited labeled tasks. From the perspective of data resources, feature transfer based on transfer learning cannot encompass the entire fault dataset and mine useful information of unlabeled data, which causes a certain waste of the available information resources.

Taking the considerable fault information of unlabeled data into account, which is the most inexpensive data available in industrial scenarios, designing a semi-supervised algorithm structure appears to be a viable solution to address the issue above. The graph-based semi-supervised learning method [11,12,13] constructed a graph structure by regarding samples as vertices and regarding the similarity between points as edges, and thus the attribution of labeled samples could be propagated to unlabeled samples due to the hierarchy structure. To fully use the more abundant unlabeled data, Wu et al. [14] designed a hybrid classification autoencoder as a one-input two-output configuration consisting of the reconstruction of the input and the prediction of the health condition. Analogously, encoder–decoder network architectures based on CNNs [15] and LSTM [16] are established to distinguish the abnormal regime from the normal operating regimes by the magnitude of the reconstruction loss. As is common practice, a skipped connection was introduced in the encoder–decoder architectures, which was known as a vanilla ladder network (LAN) [17]. The vanilla LAN constantly varied in the backbone based on a typical deep learning algorithm to obtain higher training efficiency [15,18]. Zhang et al. [19] established two independent variational autoencoder (VAE)-based deep generative models to obtain the low-dimension latent features for labeled and unlabeled data, respectively. Accordingly, the multi-channel structure enabled the semi-supervised network to learn the fault representation of both labeled and unlabeled data.

Regarding aspects of the data augmentation-based strategy, some researchers attempt to extract more sensitive fault features based on signal processing. Zhang et al. [20] input the time-frequency wavelet coefficients into a multiple association layers network combining LAN and a variational autoencoder with less-labeled samples. Roozbeh et al. [21] fused the information of the raw sensory measurements in three different domains, and Yu et al. [22] employed seven data augmentation strategies. However, the tremendous data preprocessing procedure ignores the end-to-end feature extraction ability of deep learning. Furthermore, to alleviate the limited labeled problem, generating data with the same distribution of labeled data is regarded as an intuitive solution [8]. Ding et al. [23] utilized the probabilistic mixture model and the Markov Chain Monte Carlo algorithm to expand the fault dataset, which could provide large amounts of fake data. Tao et al. [24] generated pseudo-cluster labels for labeled and unlabeled data by adopting density peak clustering strategies. In addition, deep generative models were often utilized to generate new samples for labeled minority fault samples, such as GAN [25,26,27,28] and VAE [29,30]. Difficulties arise, however, when the quality of generated samples should be ensured to implement the data augmentation-based strategies.

Taken together, the research described above has the following shortcomings when facing the lack of labeled data and the variable working conditions:

1. These two challenges are usually overcome individually, and few works in the literature have studied these two issues simultaneously.
2. Closer attention is paid to expanding labeled data for supervised learning, while considerable fault information contained in unlabeled data is ignored and wasted.
3. More than ten labeled training samples are chiefly required; however, the available labeled samples are fewer in real industrial scenarios.

Recent advances in face recognition are attributed to the rise of metric learning. Unlike generative networks, which need to pay attention to each detail of the labeled data distribution, metric learning shows its promising potential to learn discriminative embeddings that can distinguish from other samples. Typically, contrastive loss [31] and triplet loss [32] could group intra-class samples closely while pushing inter-class samples distantly in the embedding space of pairwise samples. The contrastive loss could be introduced as a regularization [33,34,35] to learn working condition-independent features. Rombach et al. [36] considered triplets of training samples and learned invariance representation in the context of changing operations. Customarily, the hard example mining strategy [37,38] is often integrated with triplet loss to enhance the representation learning ability for the later network training stage. As a result, it provides the possibility of mining limited labeled data, which lays emphasis on the similarity among pairwise samples in the embedding space, and is able to learn fault-related rather than working condition-related representation.

Given the shortcomings of the above methods and enlightened by metric learning, both algorithm structure-level and feature-level aspects are considered in this paper. In terms of the algorithm’s structure-level, a CNN-based ladder network (CLAN) with path interaction is established to extract features from the most readily available unlabeled data and the limited labeled data. From the aspects of the feature-level, the similarity among anchor, positive and negative samples are calculated in the embedding space based on metric learning, in which extremely limited labeled samples can be regarded as hard samples to mine fault-related information and eliminate the working condition shifting effect. Therefore, the acquired classification error, reconstruction error and triplet loss are jointly defined as the objective function for the proposed method. The main contributions of this study, as well as the acquisition of the objective function, are listed as follows:

1. CLAN, a novel CNN-based ladder network, replaces the vanilla ladder network (LAN) backbone with a CNN and integrates the structure of the vanilla ladder network. Thus, the classification error of labeled samples and the reconstruction error of unlabeled samples can be obtained, and the parameters of the training process can be reduced by a simplified combination activation function and a path-interaction strategy.
2. To further alleviate the feature distribution shifting problem under variable working conditions, the triplet loss with the hard sample mining strategy is utilized to enlarge the margin among the embeddings of the limited labeled samples under different working conditions, which enables the proposed method to emphasize the fault-related features.
3. The proposed method is evaluated on two datasets: the first is the public bearing dataset from Case Western Reserve University (CWRU) for comparison with other state-of-the-art algorithms and the second is the experimental bearing dataset from our laboratory test rig of the motorized spindle to illustrate its extensive applicability. A few labeled data are selected randomly to verify the effectiveness of the proposed method. Moreover, variable working conditions are able to prove the ability of the learning distribution-invariant features.

The remaining part of the paper is organized as follows. In Section 2, the theoretical background is expounded. Section 3 concentrates on introducing the details of the proposed method. In Section 4, three case studies are given to illustrate the accuracy and robustness of the proposed method for extremely limited labeled samples under variable working conditions. Finally, Section 5 concludes this work and gives direction for future work.

## 2. Primary Theoretical Background of the Proposed Method

### 2.1. Semi-Supervised LAN

In the field of unsupervised learning, a two-stage strategy of “unsupervised pre-training + supervised fine-tuning” [39] is adopted; however, the two stages are independent from each other. Semi-supervised learning is an amalgamation of supervised and unsupervised learning. The unsupervised learning part can retain the original data information to the greatest extent through data reconstruction, while the supervised learning part attempts to keep the task-related information. Given this, to make the supervised learning compatible with the unsupervised learning, a specific structure is designed for the semi-supervised LAN which provides fault information extraction paths for both labeled and unlabeled data, respectively.

**Figure 1:**
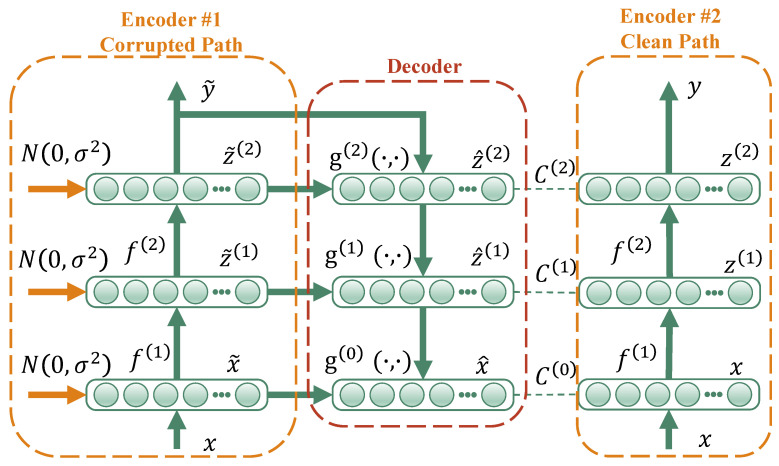
A conceptual illustration of the vanilla LAN.

The structure of the vanilla LAN was proposed by Rasmus et al. [17], which consisted of a corrupted encoder path inspired by the denoising autoencoder [40], a decoder path for data reconstruction and a clean encoder path, as shown in Figure 1. In particular, the skipped connections between the corrupted encoder path and the decoder path enable the model to put emphasis on classification-relevant features at higher layers, while the original data information is ensured to transmit to the decoder path for data reconstruction. A cardinal principle of the vanilla LAN is described as follows.

Given a sequence of sample set $X={\left\{x^{i}\right\}}_{i=1}^{M}\in {ℜ}^{1\times D}$, let M denote the number of samples and D denote the length of a sample. According to whether there is a label, the sample set is further divided into a labeled dataset $X^{L}={\left\{x^{i},y^{i}\right\}}_{i=1}^{N}\in {ℜ}^{1\times D}$ and an unlabeled dataset $X^{U}={\left\{x^{i}\right\}}_{i=N+1}^{M}\in {ℜ}^{1\times D}$, where $y^{i}\in \{1,2,3\ldots \}$ is the label for the labeled dataset, and N denotes the number of labeled samples, N≪M.

Generally, the vanilla LAN is based on a fully connected autoencoder network, and batch normalization (*BN*) and rectified linear units (*ReLU*) are applied to each layer, including the top-level layer. The corrupted noise ${\left\{\varepsilon_{l}\right\}}_{l=1}^{L}$ obeying the Gaussian distribution is implemented for the labeled samples, which is an auxiliary task to denoise representations at every level. Thus, the supervised classification cost $C_{c}$ of the noisy output $\tilde{y}$ and $y^{i}$ can be obtained as Equation (1).

(1) $$C_{c}=-\frac{1}{N}\sum_{i=1}^{N}\log P\left(\tilde{y}=y^{i}|x^{i}\right),$$

As for the unlabeled samples, a ladder-shaped encoder–decoder architecture provides an additional target with a data reconstruction error. More specifically, fault representations of each layer can be obtained through the corrupted encoder path, and then the top-level fault representation is fed into the decoder path in reverse order. Formally, the operation can be expressed as follows:

(2) $$\tilde{x},{\tilde{z}}^{\left(1\right)},\ldots ,{\tilde{z}}^{\left(L\right)}={\mathrm{Encoder}}_{corrupted}{\left(x\right)}_{\;},$$

(3) $$\hat{x},{\hat{z}}^{\left(1\right)},\ldots ,{\hat{z}}^{\left(L-1\right)}=Decoder\left({\tilde{z}}^{\left(1\right)},{\tilde{z}}^{\left(2\right)},\ldots ,{\tilde{z}}^{\left(L\right)}\right),$$

where ${\mathrm{Encoder}}_{corrupted}{\left(\cdot \right)}_{\;}$ and $Decoder\left(\cdot \right)$ are the fully connected autoencoder network, and x, $\tilde{x}$, $\hat{x}$, ${\tilde{z}}^{\left(L\right)}$ are the input, the corrupted input, the reconstructed input and the top-level fault representation, respectively. The variables ${\tilde{z}}^{\left(l\right)}$ and ${\hat{z}}^{\left(l\right)}$ are the corrupted and the reconstructed fault representation in hidden layer l.

To incorporate the information of the upper layer and the skipped connection in the decoder path, a combinator activation function $g\left(\cdot ,\cdot \right)$ is designed in an element-wise manner, as expressed in Equation (4).

(4) $${\hat{z}}^{\left(l\right)}=g\left({\tilde{z}}^{\left(l\right)},u^{\left(l+1\right)}\right),$$

where $u^{\left(l+1\right)}$ is the vertical fault representation learned from the upper layer. To provide the encoder–decoder architecture with clean reconstruction targets, the unlabeled samples are fed in the clean encoder path, which has a similar operation with $Encoder_{corrupted}$ but without the Gaussian noise:

(5) $$x,z^{\left(1\right)},\ldots ,z^{\left(L\right)}=Encoder_{clean}\left(x\right),$$

where $z^{\left(l\right)}$ represents the fault representation learned in hidden layer l. Thus, the unsupervised reconstruction costs can be obtained:

(6) $$C_{R}=ReconsCost\left(z^{\left(l\right)},{\hat{z}}^{\left(l\right)}\right),$$

where $ReconsCost\left(\cdot \right)$ is in terms of square error.

The final objective function is a weighted sum of $C_{c}$ and $C_{U}$, as expressed in Equation (7), which is trained by backpropagation to assist the supervised learning by adding unsupervised tasks.

(7) $$\begin{array}{ll} \;Cost & =-\lambda_{C}\sum_{n=1}^{N}\log P\left(\tilde{y}=y\left(n\right)|x\left(n\right)\right)\\ & +\lambda_{R}\sum_{n=n+1}^{M}\sum_{l=1}^{L}\lambda_{l}ReconsCost\left(z^{\left(l\right)}\left(n\right),{\hat{z}}^{\left(l\right)}\left(n\right)\right) \end{array},$$

where $\lambda_{C}$ and $\lambda_{R}$ are the weight for the supervised classification cost and the unsupervised reconstruction costs. $\lambda_{l}$ denotes a layer-wise hyperparameter to determine the importance of the denoising cost in each layer.

### 2.2. Triplet Loss

**Figure 2:**
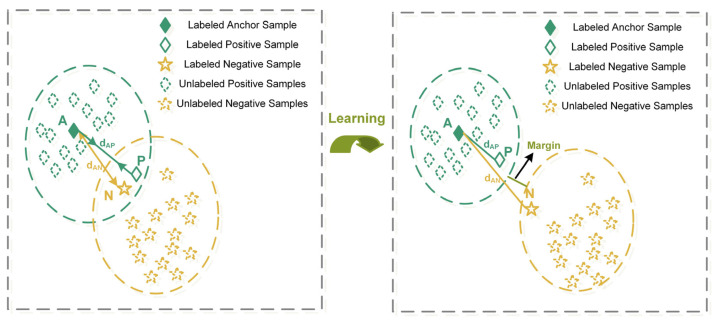
A conceptual illustration of the triplet loss.

Triplet loss is initially proposed in face recognition tasks [32], which introduces the concept of positive and negative samples to learn representations in the embedding space. Superior to the predetermined categories of the *SoftMax* function, triplet loss pays close attention to learning a mapping of a Euclidean space where distances directly correspond to a measure of the pairwise samples. As shown in Figure 2, the triplet samples consist of an anchor sample $x_{i}^{a}$, a positive sample $x_{i}^{p}$ and a negative sample $x_{i}^{n}$. The triplet loss attempts to enforce a margin between different categories and to group samples from the same category according to the distribution discrepancy. Thus, the following condition needs to be met:

(8) $$\begin{array}{c} {\left\|f\left(x_{i}^{a}\right)-f\left(x_{i}^{p}\right)\right\|}_{2}^{2}+\alpha <{\left\|f\left(x_{i}^{a}\right)-f\left(x_{i}^{n}\right)\right\|}_{2}^{2},\\ \forall \left(f\left(x_{i}^{a}\right),f\left(x_{i}^{p}\right),f\left(x_{i}^{n}\right)\right)\in \Gamma \end{array},$$

where $f\left(\cdot \right)$ is a mapping function for Euclidean space, α is the margin maintained between classes and Γ includes all possible triplets of the samples. CNN, a deep network architecture, is widely used to minimize the loss $L_{tri}$:

(9) $$L_{Tri}=\sum_{\underset{y_{a}=y_{p}\neq y_{n}}{a,p,n}}\max\left({\left\|f\left(x_{i}^{a}\right)-f\left(x_{i}^{p}\right)\right\|}_{2}^{2}-{\left\|f\left(x_{i}^{a}\right)-f\left(x_{i}^{n}\right)\right\|}_{2}^{2}+\alpha ,0\right),$$

## 3. The Proposed Method

### 3.1. An Overview of the Proposed Method

**Figure 3:**
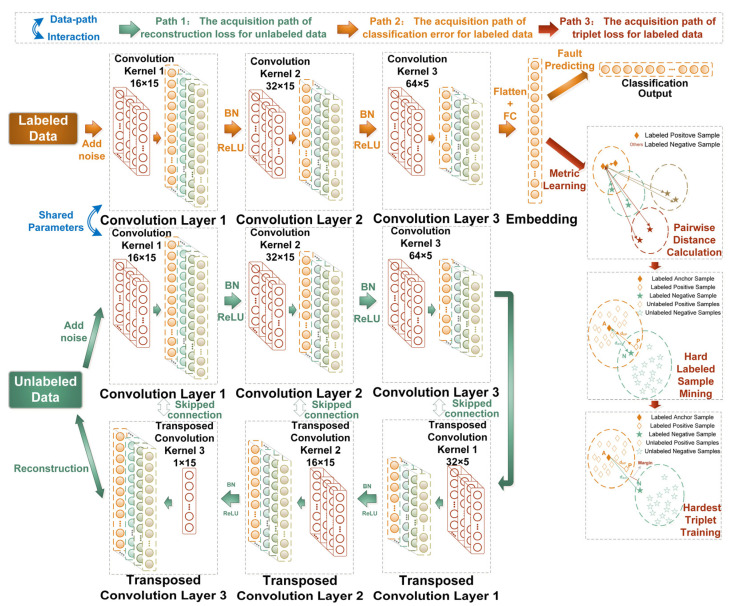
The architecture of the proposed method.

In this work, we innovatively proposed a triplet-guided path-interaction CNN-based ladder network (Tri-CLAN) as a semi-supervised model for extremely limited labeled samples under variable working conditions. Compared to conventional semi-supervised deep learning methods, the innovation of Tri-CLAN is mainly reflected in two aspects: on the one hand, a CNN is utilized to substitute for the fully connected (FC) layer in the vanilla LAN, and reasonable model simplification is implemented to prevent over-fitting; on the other hand, triplet loss is introduced to further mine hard samples from limited labeled data, which forces the model to learn distinctive and working condition-independent embeddings among samples. The architecture of Tri-CLAN is displayed in Figure 3, and the three acquisition paths of the major losses are described in the following subsections.

### 3.2. Reconstruction Loss for Unlabeled Data

**Figure 4:**
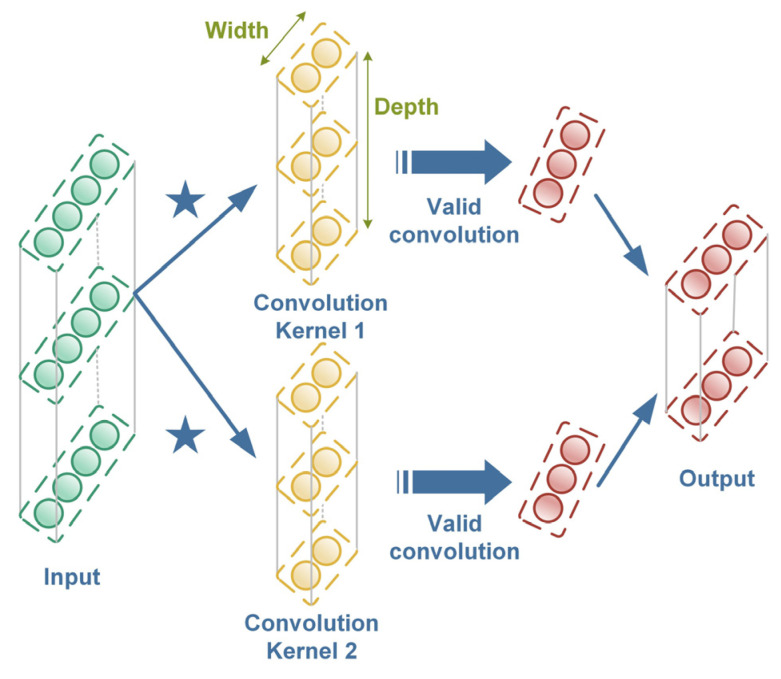
Demonstration of the 1D convolution operation.

As depicted in Path 1 of Figure 3, the acquisition path of reconstruction loss for unlabeled data is composed of a corrupted encoder and a decoder. In the corrupted encoder, the Gaussian noise ε with mean 0 and variance are injected into the unlabeled samples $X^{U}={\left\{x^{i}\right\}}_{i=N+1}^{M}$ to learn internal stable representation and resist the noise perturbation, which originates from the vanilla ladder network. Specifically, standard Gaussian noise with mean 0 and variance 1 is selected because of its symmetrical characteristic and the consistence with the data distribution after batch normalization. The corrupted representation ${\tilde{z}}^{\left(l\right)}$ can be obtained by the following operation:

(10) $${\tilde{z}}^{\left(0\right)},{\tilde{z}}^{\left(1\right)},\ldots ,{\tilde{z}}^{\left(L\right)}={\mathrm{Encoder}}_{corrupted}\left(X^{U}\right),$$

where ${\mathrm{Encoder}}_{corrupted}\left(\cdot \right)$ denotes the 1D convolution operation *Conv(∙)* followed by the batch normalization operation *BN(∙)* and the non-linear activation operation *ReLU(∙)* as follows:

(11) $${\tilde{Z}}^{\left(l\right)}={\tilde{h}}^{\left(l\right)}=X^{U}+\varepsilon ,l=0,$$

(12) $${\tilde{z}}_{\mathrm{pre}}^{\left(l\right)}=Conv\left({\tilde{h}}^{\left(l-1\right)}\right),1\leq l\leq L,$$

(13) $${\tilde{z}}^{\left(l\right)}=BN\left({\tilde{z}}_{pre}^{\left(l\right)}\right),$$

(14) $${\tilde{h}}^{\left(l\right)}=ReLU\left(\gamma^{\left(l\right)}\times \left({\tilde{z}}^{\left(l\right)}+\beta^{\left(l\right)}\right)\right),1\leq l\leq L,$$

where ${\tilde{z}}_{pre}^{\left(l\right)}$, ${\tilde{h}}^{\left(l\right)}$ stand for the intermediate variables at layer l and $\gamma^{\left(l\right)}$ and $\beta^{\left(l\right)}$ are the scaling and offset coefficients of the batch normalization operation, respectively. To be specific, *Conv(∙)* multiplies the local input data by the shared weight of the convolution kernel, and thus the feature matrix can be obtained and the corresponding output of *Conv(∙)* at layer l can be described as:

(15) $$\;{\mathrm{Conv}}_{output}=b_{i}^{\left(l\right)}+W_{i}^{\left(l\right)}⋆X^{\left(l^{j}\right)}=\sum_{k=1}^{K}W_{i}^{\left(l^{k}\right)}X^{\left(l^{j+k}\right)},$$

where ⋆ is the valid cross-correlation operator, $X^{\left(l^{j}\right)}$ denotes the jth local data to be convoluted, $W_{i}^{\left(l\right)}$ and $b_{i}^{\left(l\right)}$ are the weight and bias of the ith convolution kernel, K is the width of the convolution kernel and the detailed operation process of the 1D convolution operation is shown in Figure 4. Additionally, *BN(∙)* is represented by $BN(x)=\frac{x-mean(x)}{\sqrt{\mathrm{var}(x)}}$, and *ReLU(∙)* is formulated by ReLU(x)=max(0,x).

In the decoder, the corrupted representation ${\tilde{z}}^{\left(L\right)}$ at each level and the top-level representation ${\tilde{h}}^{\left(L\right)}$ of the corrupted encoder are taken as the input by introducing the skipped connection; then, sufficient fault-related information can be reserved for classification and the original low-level data can still flow into the decoder path. Thus, the contradictions between supervised fault classification and unsupervised data reconstruction can be resolved. With this strategy, the denoising representation ${\hat{z}}^{\left(L\right)}$ can be calculated by combining the corrupted representation ${\tilde{z}}^{\left(L\right)}$ from the corresponding layer of the corrupted encoder and ${\hat{z}}^{\left(l+1\right)}$ from the upper layer, as expressed by:

(16) $$\;{\hat{z}}^{\left(0\right)},{\hat{z}}^{\left(1\right)},\ldots ,{\hat{z}}^{\left(L\right)}=Decoder\left(\left({\tilde{z}}^{\left(0\right)},{\hat{z}}^{\left(1\right)}\right),\left({\tilde{z}}^{\left(1\right)},{\hat{z}}^{\left(2\right)}\right),\ldots ,\left({\tilde{z}}^{\left(L\right)},{\tilde{h}}^{\left(L\right)}\right)\right),$$

where $Decoder\left(\cdot \right)$ denotes the 1D transposed convolution operation $TransConv\left(\cdot \right)$ followed by the batch normalization operation *BN(∙)* and an element-wise activation operation $g\left(\cdot ,\cdot \right)$ as follows:

(17) $$u_{\left(l\right)}^{\mathrm{pre}}=\left\{\begin{array}{c} {\tilde{h}}^{\left(L\right)},\;\;\;\;\mathrm{if}\;l=L\\ TransConv\left({\hat{z}}^{\left(l+1\right)}\right),1\leq l<L \end{array}\right.,$$

(18) $$u^{\left(l\right)}=BN\left(u_{\left(l\right)}^{\mathrm{pre}}\right),$$

(19) $${\hat{z}}^{\left(l\right)}=g\left({\tilde{z}}^{\left(l\right)},u^{\left(l\right)}\right),$$

where $u_{\left(l\right)}^{\mathrm{pre}}$ and $u^{\left(l\right)}$ stand for the intermediate variables for the denoising representation ${\hat{z}}^{\left(l\right)}$ at layer *l*, andg(⋅,⋅) is a simplified combination activation function ${\hat{z}}^{\left(l\right)}={\tilde{z}}^{\left(l\right)}+u^{\left(l\right)}$ from which internal elements participate in the operation.

So far, the corrupted representation ${\tilde{z}}^{\left(l\right)}$ and the denoising representation ${\hat{z}}^{\left(l\right)}$ at each layer are obtained with the skipped connection to reconstruct the unlabeled data $X^{U}$, and the reconstruction loss for unlabeled data can be expressed as:

(20) $$\;C_{Recon}=ReconsCost\left(z^{\left(0\right)},{\hat{z}}^{\left(0\right)}\right)={\left\|z^{\left(0\right)}-BN\left({\hat{z}}^{\left(0\right)}\right)\right\|}^{2},$$

where $ReconsCost\left(\cdot \right)$ stands for the $l^{2}$ distance between $z^{\left(0\right)}$ and ${\hat{z}}^{\left(0\right)}$. Obviously, the reconstruction errors at each layer of the vanilla LAN are reducible to an error between the input corrupted data and the reconstructed data, and thus the computation burden of the acquisition path for reconstruction error can be reduced.

### 3.3. The Classification Error for Labeled Data

The acquisition path of the classification error consists of a corrupted encoder sharing parameters with the corrupted encoder of the acquisition path for reconstruction loss, which is a branch followed by fully connected layers for the prediction of fault types, as depicted in Path 2 of Figure 3. In the corrupted encoder for labeled samples $X^{L}={\left\{x^{i},y^{i}\right\}}_{i=1}^{N}$, the embedding $embed^{\left(l\right)}$ of fully connected layers and the classification output $\tilde{y}$ can be computed as follows:

(21) $$\;embed^{\left(l\right)}=BN\left(W^{\left(l\right)}embed^{\left(l-1\right)}\right),\mathrm{if}\;\mathrm{layer}\;l\;\mathrm{is}\;a\;\mathrm{fully}\;\mathrm{connected}\;\mathrm{layer},$$

(22) $$\;\tilde{y}=SoftMax\left(embed_{i}^{\left(L\right)}\right),$$

where $W^{\left(l\right)}$ is the weight matrix of fully connected layer l, L is a classification layer and $SoftMax\left(\cdot \right)$ is represented by $SoftMax\left(x_{i}\right)=\frac{e^{x_{i}}}{\sum_{k=1}^{K}e^{x_{k}}}$. Thus, the classification error for labeled data can be expressed as the cross-entropy:

(23) $$\;C_{Class}=-\frac{1}{N}\sum_{i=1}^{N}\;\log\;P\left(\;y\;˜=y^{i}|x^{i}\right),$$

### 3.4. Triplet Loss for Labeled Data

The acquisition path of triplet loss, another branch of labeled data, is composed of pairwise distance calculation, hard labeled sample mining and hardest triplet training. From the aspect of mapping space, the fault information is mined to the greatest extent according to the similarity among triple samples. Intuitively, the large amount of possible generating triplets may result in uninformative representation, rendering a slow convergence and an overlong training duration. With this realization, selecting the hardest positive and negative samples provides an organizational modification for the triplet loss, which dramatically helps learning essential fault-related representation of limited labeled data. As depicted in Path 3 of Figure 3, all pairwise distances among each anchor sample $embed_{a}^{\left(L\right)}$, randomly sampling positive samples $embed_{p}^{\left(L\right)}$ and negative samples $embed_{n}^{\left(L\right)}$ are first calculated, and then triplets that violate the constraint in Equation (8) are selected. Eventually, the hardest triplet is trained according to Equation (24), which is the loss of learning:

(24) $$\begin{array}{ll} C_{Tri}=\sum_{i=1}^{P}\sum_{a=1}^{K} & [\underset{p=1\cdots K}{max}\left({\left\|f\left(embed_{a^{i}}^{\left(L\right)}\right)-f\left(embed_{p^{i}}^{\left(L\right)}\right)\right\|}_{2}^{2}\right)\\ & {\left.-\underset{\begin{array}{c} j=1\cdots P\\ n=1\cdots K\\ j\neq i \end{array}}{min}\left({\left\|f\left(embed_{a^{i}}^{\left(L\right)}\right)-f\left(embed_{n^{j}}^{\left(L\right)}\right)\right\|}_{2}^{2}\right)+a\right]}_{+} \end{array},$$

where P is the number of the fault classes, K is the sample number of each fault class and PK is the quantity of all labeled samples. At this point, it is essential to note that the first term represents the distance metric of the hardest positive sample, and the second term represents the distance metric of the hardest negative samples in the Euclidean space.

### 3.5. The Final Objective Function

**Figure 5:**
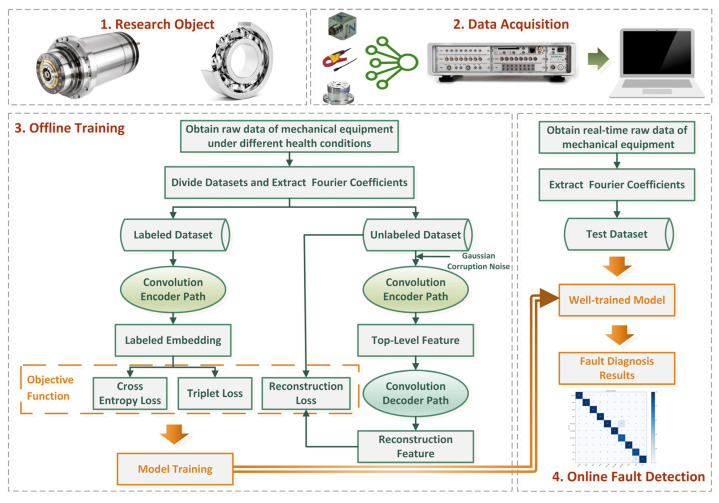
A flowchart of the proposed method.

Profiting from the joining of semi-supervised learning and metric learning, the proposed method can not only excavate discriminative features from unlabeled and labeled samples, but it can also optimize the embedding space of limited labeled samples to further obtain semantically meaningful fault features. As the illustration of the information flow shown in Figure 5 shows, the final objective function is composed of the reconstruction loss, the cross-entropy loss and the triplet loss, which can be defined as:

(25) $$\begin{array}{ll} \;C_{Total} & =C_{Recon}+C_{Class}+C_{Tri}\\ & =\frac{1}{N+M}\sum_{i=1}^{N+M}ReconsCost\left(z_{\left(i\right)}^{\left(0\right)},{\hat{z}}_{\left(i\right)}^{\left(0\right)}\right)-\frac{1}{N}\sum_{i=1}^{N}\log P\left(\tilde{y}=y^{i}|x^{i}\right)+C_{Tri} \end{array},$$

Unlike the fixed “pre-trained + fine-tuning” mode, $C_{Recon}$ and $C_{Tri}$ can both be regarded as a regularized term into the supervised classification costs, which is able to optimize the multi-path costs in a one-phase training simultaneously. Here, historical data of mechanical equipment under different health conditions is preprocessed to obtain the three losses in the offline training stage, and then the final objective function is minimized by introducing the Adam algorithm [41]. When Tri-CLAN has been well trained, the second path with the classifier is used for detecting fault types of online real-time data.

## 4. Experimental Studies

Several experiments on two case studies are conducted to evaluate the effectiveness and applicability of the proposed method. One is the public bearing dataset from CWRU, which is regarded as the standard dataset for objective comparison with the state-of-the-art algorithms, and the other is the laboratory test rig of the motorized spindle.

### 4.1. Implementation Details

In experimental studies, the raw data are usually 1D, and fast Fourier transform (FFT) is used to highlight more frequency-domain information. In order to facilitate the subsequent description of the architecture of the proposed method and the corresponding comparison methods, several basic modules are built, including a corrupted encoder module based on 1D CNN (CE-CNN), a decoder module based on 1D CNN (DE-CNN), a corrupted encoder module based on FC (CE-FC), a decoder module based on FC (DE-FC), a predicting module and a metric learning module. In the CE-CNN and DE-CNN, three 1D convolutional layers and three 1D transposed convolutional layers are separately constructed followed by *BN* and *ReLU* operation. In the CE-FC and DE-FC, a fully connected form with three hidden layers is adopted for a fair comparison. Likewise, the predicting module with FC is used for dimension reduction and fault classification. Put slightly differently, the predicting #1 requires an additional flattening operation to integrate the features of several channels into a 1D form. The detailed kernel size and neuron number of each module are listed in Table 1.

This study applies comparison to conduct a systematic and intensive study, and several deep learning methods based on basic modules are employed to verify the effectiveness of the proposed method, which contains a conventional CNN and vanilla LAN. Additionally, methods without the metric learning module are employed to discuss the constructive contribution for limited labels and variable working conditions. The detailed architecture of these methods can be shown in Table 2, and the parameter setup for experiments in case studies are listed in Table 3.

The experiments are carried out on a computer with NVIDIA Quadro RTX 6000 GPU, and PyTorch platform is implemented as the backend for programming. For the experimental error reduction, each case study experiment is repeated with ten trials to avoid contingency.

### 4.2. Case Study 1: Public Bearing Dataset of CWRU

#### 4.2.1. Fault Dataset Description

**Figure 6:**
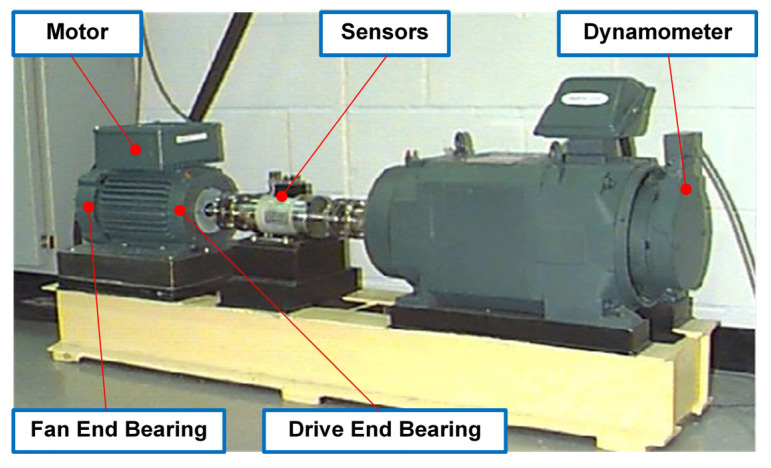
The experimental test rig of CWRU.

As is well known, the public bearing dataset of CWRU is most commonly used to verify the effectiveness of diagnostic algorithms. As shown in Figure 6, the experimental test rig of CWRU mainly consists of a 2 HP motor, a dynamometer and a connecting part with sensors, including a torque transducer and an encoder. In this study, the drive end bearing supporting the motor shaft is selected as the research object, and its vibration acceleration signal is collected using the acceleration sensor placed on the bearing pedestal at the drive end. Considering that it is often difficult to maintain a stable working condition and reach a high sampling frequency in practical industrial applications, the vibration data collected at the sampling frequency of 12 kHz under different working conditions, listed in Table 4, are selected as training data and testing data. At the same time, in order to meet the industrial requirements, it is not only necessary to identify the fault location of the research object, but also to distinguish its damage degree to provide a basis for subsequent maintenance strategies. Therefore, both training data and testing data are divided into ten categories according to the fault location and damage degree, as shown in Table 5.

Before beginning the experiments, the datasets under different working conditions need to be preprocessed uniformly, and a sample consisting of 2048 points is intercepted from the original vibration data of each fault label. Then, FFT and zero-mean normalization processing are introduced into each sample to obtain samples with a length of 1024. Hereafter, 3000 and 1000 samples are generated for training and test datasets, respectively. According to different experimental purposes, the training data will be subsequently divided into labeled training data and unlabeled training data in different proportions.

#### 4.2.2. Experiments Setup for Fault Diagnosis

Two experiments were conducted to verify the superiority of the proposed Tri-CLAN. Firstly, to preliminarily demonstrate the diagnostic performance, three datasets (A_1_, B_1_, C_1_) with different labeled sample numbers are established by randomly selecting from working condition C1; the exact number of training samples and testing samples for each category is listed in Table 6. Then, the ability of learning distribution-invariant features under variable working conditions is ulteriorly proved by building four datasets (A_2_, B_2_, C_2_, D_2_) on the foundation of experiment 1, and the number of training samples and testing samples is listed in Table 7.

#### 4.2.3. Results Analysis for Experiment 1

**Figure 7:**
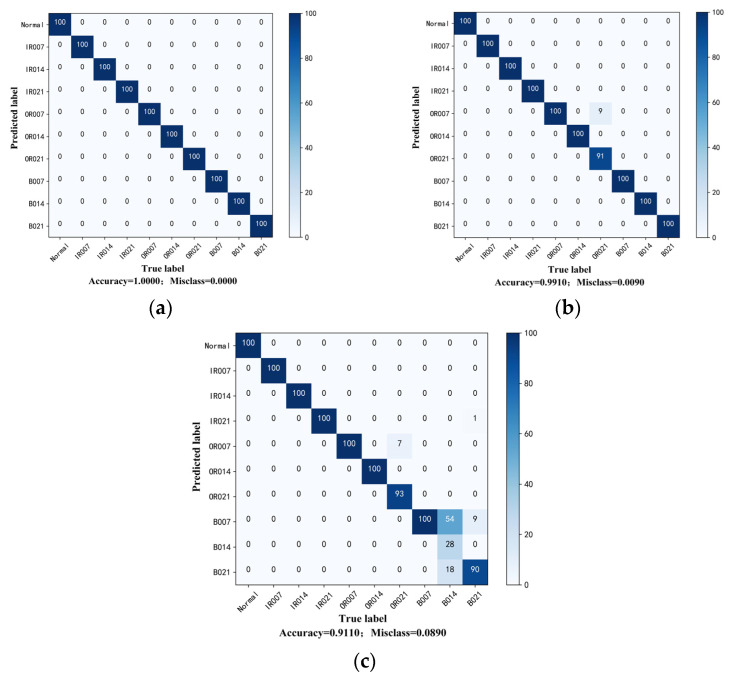
Confusion matrices for experiment 1 results on C1: (**a**) A_1_: five labels; (**b**) B_1_: two labels; (**c**) C_1_: one label.

**Figure 8:**
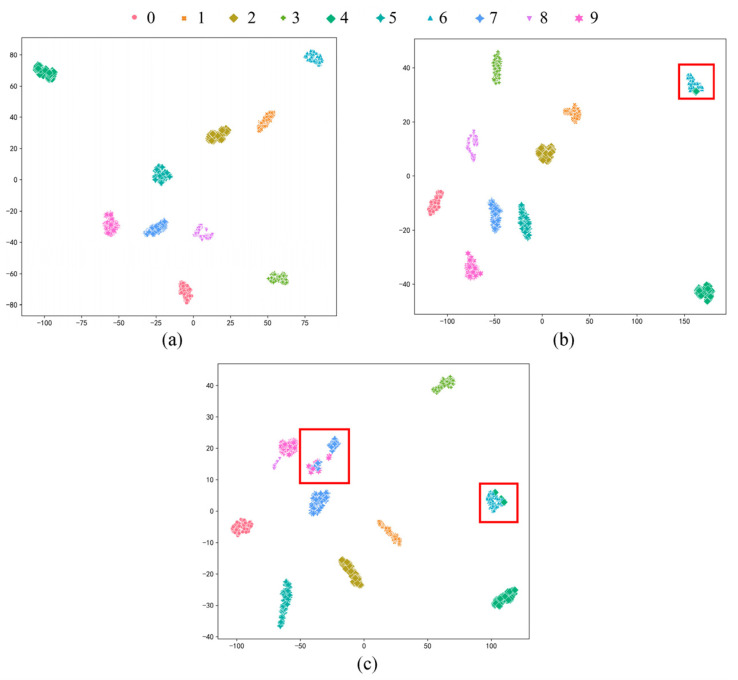
Visualization features for experiment 1 results on C1: (**a**) five labels; (**b**) two labels; (**c**) one label.

From a more specific perspective, the matching matrix between the predicted diagnosis results and the actual labels is displayed in a visual form to reflect the detailed classification, and the clustering effects of features are shown by t-distributed stochastic neighbor embedding (t-SNE). It is worth adding that the confusion matrix diagram shown in Figure 7 and the visualization features shown in Figure 8 are the diagnosis results which are closest to the average accuracy rate of the ten trials. It can be seen that the fault location and damage degree of the bearing are all correctly classified when the labeled sample number is five and the accuracy is 100%. Samples belonging to different fault categories in the feature space are distinguished clearly, and there is no overlap in the feature space. Compared with the experiment taking five labeled samples, experiments with two and one labeled samples can be regarded as extremely severe conditions, and the accuracy is 99.10% and 91.10%, respectively. Specifically, only nine test sample are wrongly classified when the labeled sample number is two, which belongs to the misclassed damage degree for bearing OR. It can also be observed from the feature space that sample features are misclassified into other categories. When the labeled sample number is one, only one sample of ball faults is predicted as OR faults, and the accuracy of fault location classification can reach 99.90%. The remaining 88 misclassed test samples are all damage degree identification errors, which are not identified as normal and which affected routine repair and maintenance of the equipment.

**Figure 9:**
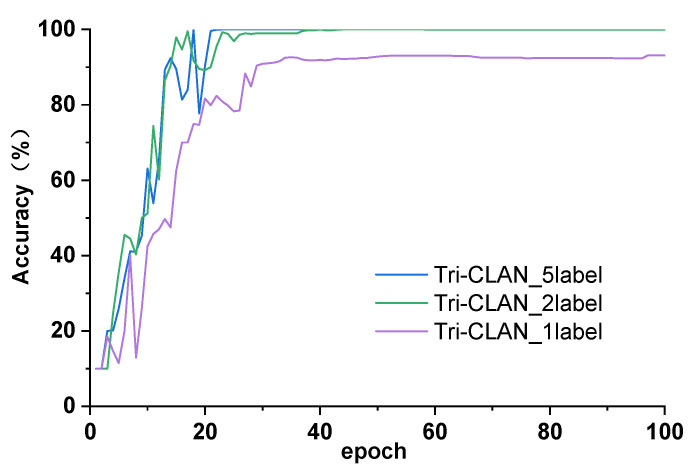
Accuracy curves for experiment 1.

As shown in Figure 9, the convergence during training iterations is analyzed for experiment 1. When the labeled sample numbers are five and two, the accuracy curve gradually trends towards smoothing after 20 iterations. By comparison, the accuracy curve with one labeled sample appears to unstably change with sharp fluctuations at the beginning, and it trends towards smoothing after 40 iterations. It is relatively hard to provide sufficient fault information due to the lack of labeled fault samples, which results in a smaller slope of the convergence curve and a lower value of the last convergence accuracy.

**Figure 10:**
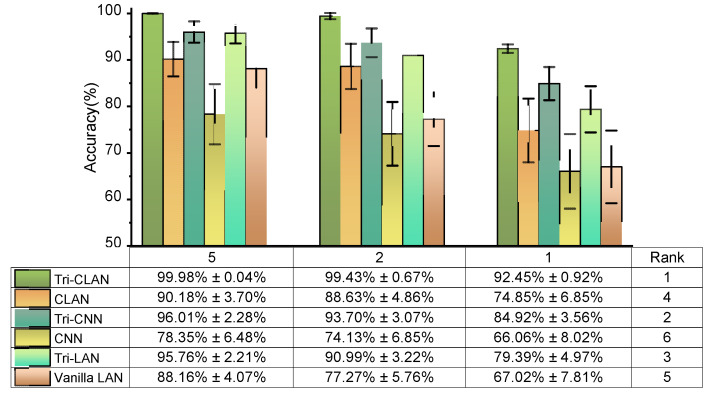
Ablation experimental results for experiment 1.

On the premise of preliminarily proving the effectiveness of the proposed algorithm with extremely limited labeled samples, we further discuss the contribution of the ladder-shaped semi-supervised network structure and triplet loss for the proposed Tri-CLAN. Therefore, the methods listed in Table 2, classic baseline (CNN), methods with ladder-shaped architecture in the form of encoder–decoder (CLAN, Tri-LAN, Vanilla LAN) and methods adding metric-learning (Tri-CNN, Tri-LAN), are established to provide a reference for analysis. In order to intuitively compare ablation experimental results, accuracy bar charts with error bands for five, two and one labeled samples are presented in Figure 10. All methods in Table 2 are ranked according to the average accuracy with standard deviation. It is evident that the accuracy of the proposed Tri-CLAN algorithm is significantly higher than that of CLAN, CNN and Vanilla LAN under various labeled sample numbers. Therefore, we only discuss the comparison among the proposed Tri-CLAN, Tri CNN and Tri LAN. When the labeled sample number is five, the accuracy of Tri-CLAN, Tri-CNN and Tri-LAN is higher than 95%, and the accuracy of Tri-CLAN is 3.97% and 4.22% higher than Tri-CNN and Tri-LAN, respectively. Moreover, the standard deviation of Tri-CLAN is close to zero; in other words, the proposed method has good robustness and its accuracy is basically stable at 100%. When the labeled sample number is two, the accuracy of Tri-CLAN can still stay above 99%, while the accuracy of Tri-CNN and Tri-LAN are reduced to less than 95%. As the case with the least labeled data, one labeled sample is a tremendous test for diagnosis algorithms to learn fault representation; however, the average accuracy of Tri-CLAN is surprisingly 92.45%. As shown in the confusion matrix above, the accuracy of fault location classification can reach 99.70%. From the perspective of standard deviation, the accuracy of the proposed Tri-CLAN basically fluctuates within 1% in the three cases of various labeled samples, while the standard deviation of the Tri-CNN and Tri-LAN is greater than 2% even in the case of five labeled samples.

**Figure 11:**
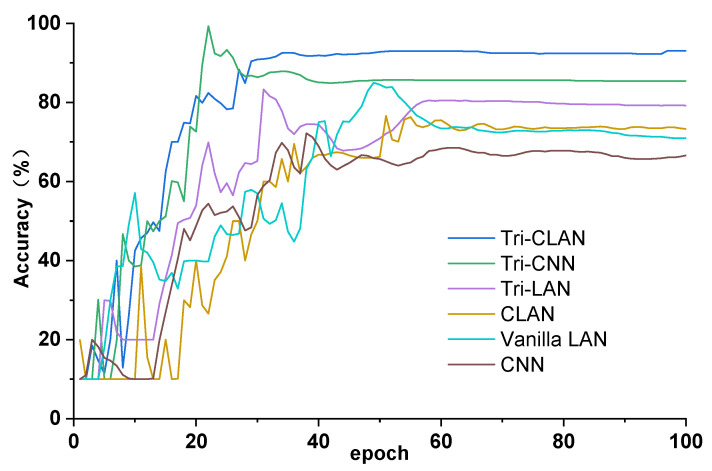
Accuracy curves of ablation experiment with 1 labeled sample.

According to the ranking in Figure 10 and the accuracy curves in Figure 11, methods adding a metric learning module generally achieve relatively high accuracy and early convergence, such as Tri-CLAN, Tri-CNN and Tri-LAN. It follows that the triplet loss plays a decisive role in the contribution to diagnosis accuracy. Subsequently, the method based on the CNN has higher accuracy and smaller fluctuation than that based on the autoencoder, and we believe that this phenomenon is related to the excellent generalization capability of the shared convolutional kernel. From the perspective of algorithm structure, the ladder-shaped encoder–decoder architecture is able to exploit an enormous quantity of unlabeled data which is usually ignored, and thus the actual distribution of each fault can be obtained. As a comprehensive combination of the triplet loss, the ladder-shaped encoder–decoder architecture and the CNN-based backbone, the proposed Tri-CLAN can deservedly learn effective fault representations and improve the accuracy with extremely limited labeled samples.

Based on the comparisons of architectures with different modules, we systematically select some excellent references from recent years with the same diagnostic purpose and compared the experimental results with the proposed Tri-CLAN in this paper. The classification category, the number of training samples and the test accuracy of the comparative experiment are all listed in Table 8. Obviously, these methods have achieved good fault identification results with few labels; for instance, the experimental setup in reference [42] is basically consistent with the experiment with five labeled samples in this paper, with an average accuracy of 98.40%. However, it can be found that the misclassed test samples are wrongly divided to other fault locations by observing the confusion matrix results in reference [42]. References [43,44] only classified fault locations and not the damage degree with an average accuracy about 90%, and we analyzed that it is bound up with the failure to utilize the unlabeled data as auxiliary.

#### 4.2.4. Results Analysis for Experiment 2

On the whole, experiment 1 focuses on the performance comparison of limited labeled samples, and from the above discussion of the experimental results, it can be proved that the proposed Tri-CLAN can effectively utilize the unlabeled samples to assist extremely limited labeled samples and improve the accuracy of the method. In a follow-up experiment, we decided to increase the difficulty of experiment 1 by training with extremely limited labeled samples under one working condition and testing under other working conditions. Herein, the actual manufacturing operation is simulated, in which it is difficult to obtain data under the same working condition.

**Figure 12:**
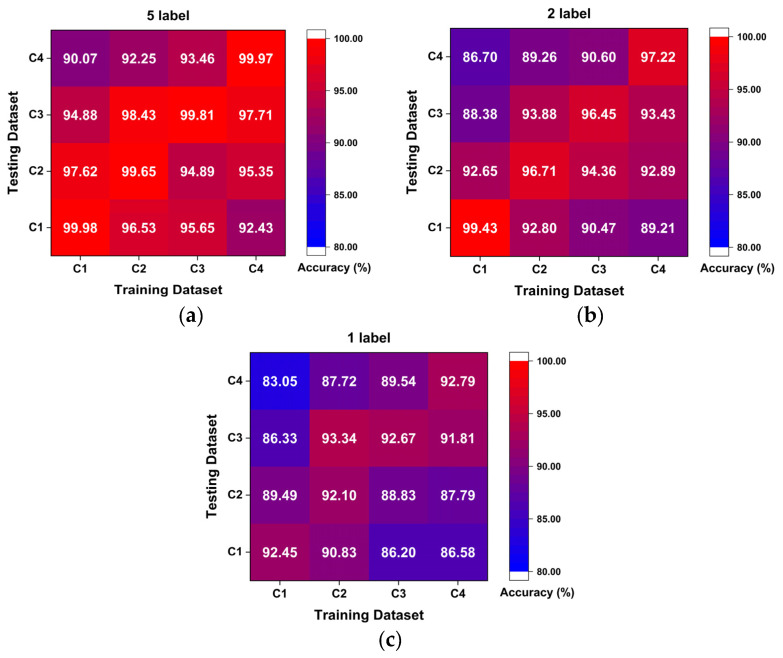
Heatmaps of experiment 2 results: (**a**) five label; (**b**) two label; (**c**) one label.

To better understand the working condition shifting effect with extremely limited samples, Figure 12 shows the results of experiment 2 in the form of heatmaps. It clearly shows the accuracy distribution by displaying different colors; broadly speaking, the accuracy of the diagnosis results is relatively reduced with the decrease of labeled samples. Secondly, a specific example under different working conditions with five labeled samples is taken for detailed discussion, as shown in Figure 12a. As a matter of course, the diagnosis results of the same working condition are higher than that of different working conditions, which can reach more than 99.50% universally. As the difference between training and testing conditions becomes larger, the accuracy rate appears to have a significant downtrend because of the difference between the probability distributions in the representation space. Another interesting phenomenon appears when tasks between different working conditions have the same difference degree, such as C1 → C4 and C4 → C1, and the diagnosis task from low-speed to high-speed shows relatively higher accuracy. It is noted that the load under different working conditions only affects the speed of the motor, and there is no mechanism to convert the torque load into the radial load on the bearing [45]. As the rotation speed decreases, the amplitude of the vibration signal becomes larger, and thus the vibration caused by faults can be highlighted and more fault representations can be learned relatively. Therefore, the fault representations learned from the low-speed C4 are sufficient to support the robust diagnostic accuracy for test under the high-speed C1. Instead, the model trained under C1 with a high rotating speed cannot afford to provide sufficient fault representations for C4 and achieve an ideal diagnosis. Similarly, this interesting phenomenon also appears in tasks under different working conditions with two and one labeled samples.

**Figure 13:**
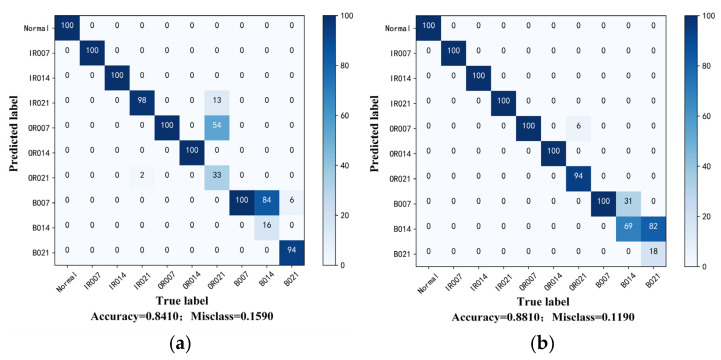
Confusion matrices of experiment 2 results with one label: (**a**) C1–C4; (**b**) C4–C1.

**Figure 14:**
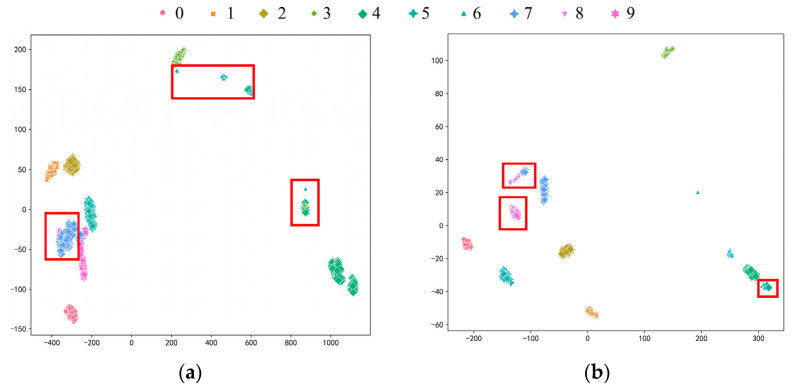
Visualization features of experiment 2 results with one label: (**a**) C1–C4; (**b**) C4–C1.

Probing into the experimental group with the most significant difference degree and the least labeled sample is meaningful to prove the effectiveness and ability for learning distribution-invariant features of the proposed Tri-CLAN. As can be seen from the above heatmap, the average accuracy of C1 → C4 and C4 → C1 is 83.05% and 86.58%, respectively, which are the worst diagnosis test results in experiment 2. To further observe the specific situation of the classification, confusion matrices and visualization features which are closest to the average accuracy are shown in Figure 13 and Figure 14, respectively. It can be seen that 15 samples are misclassified as other fault locations in the C1 → C4 experiment, and the remaining samples belong to the damage degree identification error. The features of different fault locations appear in a clear clustering state, and only a few parts among samples of different damage degrees overlap in the feature space. All misclassified samples belong to the damage degree identification error in the C4 → C1 experiment, and all samples with different fault locations can be well distinguished in the feature space. Therefore, the proposed Tri-CLAN has achieved satisfactory diagnostic results with extremely limited labeled samples under variable working conditions.

**Figure 15:**
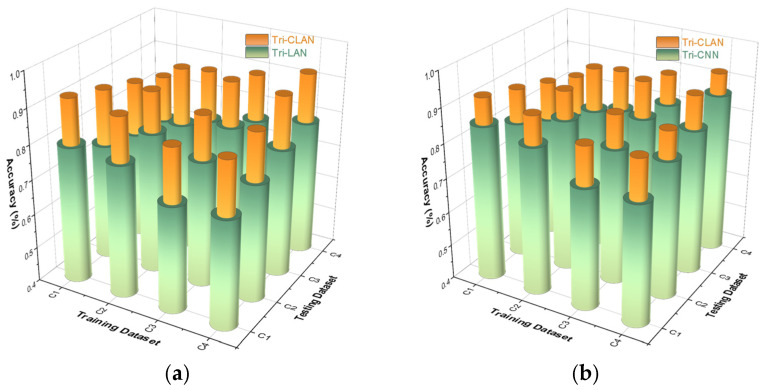
Three-dimensional histograms of experiment 2 results for comparison: (**a**) is the comparison with Tri-LAN; (**b**) is the comparison with Tri-CNN.

We review experiment 1 and prove that triplet loss plays a crucial role when the labeled samples are extremely few. To reduce unnecessary comparisons, we directly compare the proposed Tri-CLAN with the top two algorithms in experiment 1, which are the methods adding metric-learning (Tri-CNN, Tri-LAN). The comparative experimental results are presented in the form of three-dimensional histograms in Figure 15. It is clear that the proposed Tri-CLAN performs the best in experiment 2, which primarily depends on the distinctive structure. Making a concrete analysis, the ladder-shaped encoder–decoder module can obtain more unsupervised data auxiliary information and the CNN-backbone prevents overfitting, which remedies the feature distribution shifting problem and improves the overall test accuracy.

### 4.3. Case Study 2: Motor Fault Dataset of SZTU

#### 4.3.1. Fault Dataset Description

**Figure 16:**
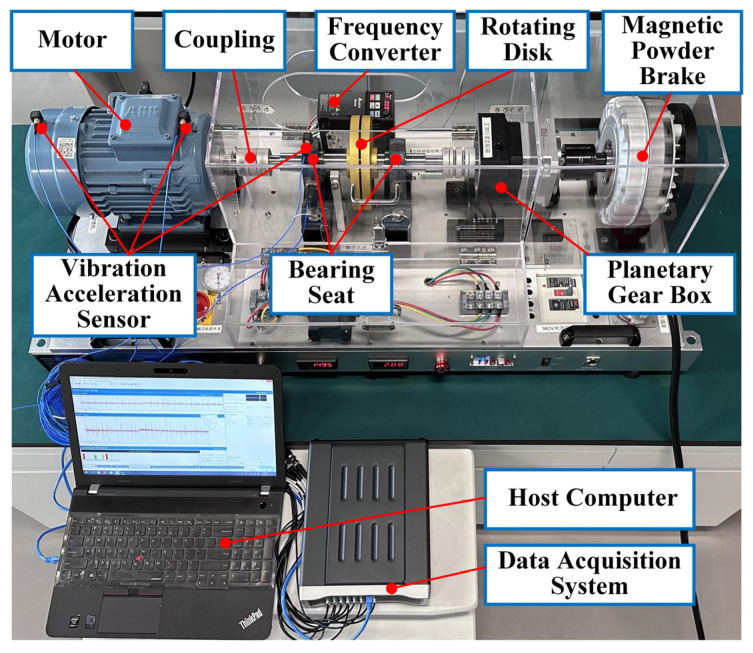
Rotating machinery fault test system.

The motor fault dataset is collected and organized by Shenzhen Technology University (SZTU). The test system is shown in Figure 16, which mainly consists of a three-phase asynchronous motor, two bearing seats, a rotating disc, a planetary gear box, a frequency converter and a magnetic powder brake. The rotating components are connected through couplings, and the vibration of the test system is obtained through a sequence of vibration acceleration sensors, a data acquisition system and a host computer. The motor speed is controlled by a frequency converter and can be adjusted within the range of 0–1750 rpm. The load of the motor is adjusted through a magnetic particle brake, which can provide a torque load of 0–50 N∙m.

In order to verify the diagnostic performance of the proposed Tri-CLAN under different working conditions, four working conditions are listed in Table 9. Especially, C1 and C3 working conditions are set to the rated speed of the motor, and C1 and C4 working conditions are set to the highest torque load. The motor fault dataset includes six fault categories and one health category, for which the corresponding labels are listed in Table 10. The vibration data at the 12 o’clock direction of the motor fan end is selected, and each 2048 sampling points are set as one sample. The sample number of the training dataset and the testing dataset are 300 and 100 for each category. Then FFT and zero-mean normalization processing are introduced into each sample. The number of training and testing samples for each category under different operating conditions is listed in Table 11, which includes labeled samples and unlabeled samples.

#### 4.3.2. Results Analysis

**Figure 17:**
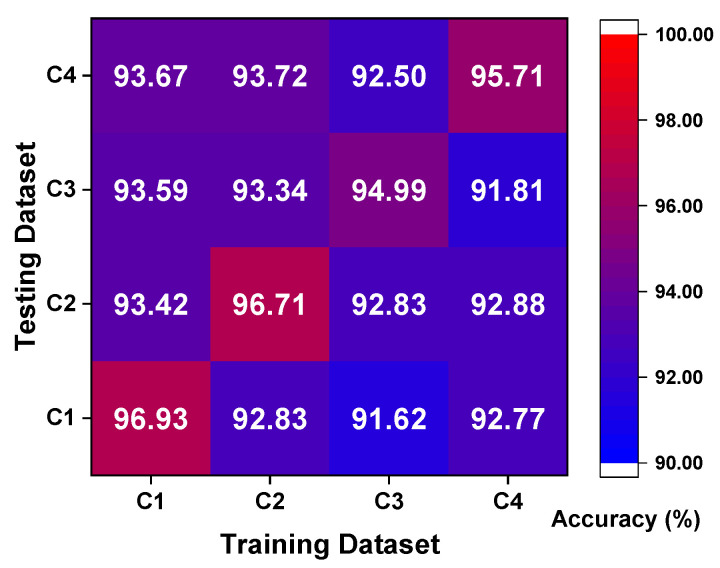
Heatmaps of motor experimental results with one label.

**Figure 18:**
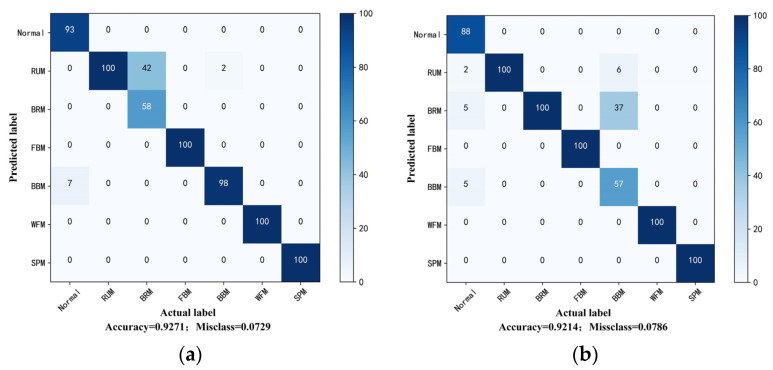
Confusion matrices for cross-working condition task: (**a**) C3–C4; (**b**) C4–C3.

We directly select one labeled sample for experimental verification under variable conditions, which is the most extreme case. The heatmaps of experimental results for one labeled sample are displayed in Figure 17. It can be seen that the result of the classification task can maintain around 95% under the same working condition. Faced with variable working conditions with one labeled sample, the classification task of fault location can still reach more than 90%. C1 and C3 working conditions have the greatest difference in both speed and load, and Figure 18 shows the confusion matrices of the cross-working condition task. It can be observed that BRM and BBM are easily misclassified among the seven fault categories. It is worth noting that none of the fault samples are misclassified as healthy samples, which can ensure the safe operation of the motor.

Compared to the publicly available CWRU dataset, the motor fault dataset has more fault categories and more complex working conditions, which can sufficiently demonstrate the applicability of the proposed method in more complex practical scenarios.

### 4.4. Case Study 3: Laboratory Bearing Dataset of Motorized Spindle Test Rig

#### 4.4.1. Fault Dataset Description and Experiment Setup

**Figure 19:**
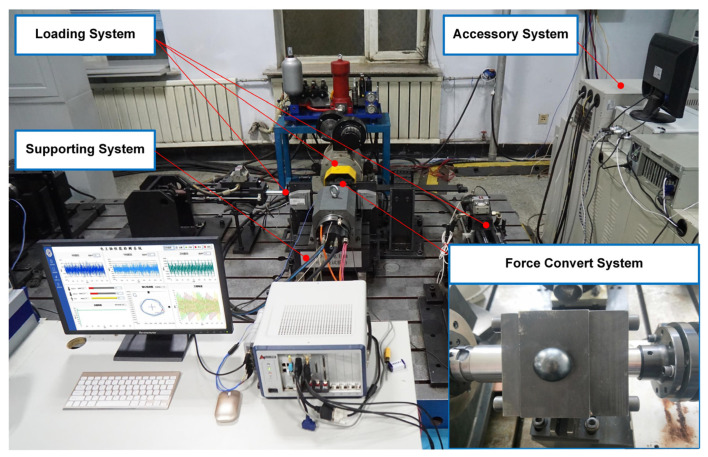
The laboratory test rig of the motorized spindle.

Here, to further illustrate the extensive applicability of the proposed method, a laboratory bearing of the motorized spindle test rig (MSTR) [46] is separately conducted to acquire data and analysis. The overall test rig is illustrated in Figure 19, which consists of a supporting system, a loading system, a force convert system and an accessory system. The bearing required for experimental verification is the core part of the force convert system, which needs to bear dynamic loads during the high-speed rotation test of the motorized spindle, such as radial load, axial load and torque load. Therefore, the bearing under dynamic load and high-speed rotation is the basis for ensuring the safe and stable operation of the reliability test of the motorized spindle. In order to ensure the accurate application of load and the safety of the experimenter, it is necessary to identify the health state of the bearing. As the research object of this case study, the vibration acceleration signal of the bearing is collected by the acceleration sensor placed on the force convert system sleeve.

To align with the experiment of case study 1, the sampling frequency of the vibration data is set to 12 kHz. The rotation speed of the bearing is consistent with that of the motorized spindle, and two working conditions with a large difference are selected for experimental verification according to the reliability test conditions in the literature [46], which are 5000 rpm (C1) and 1000 rpm (C2), respectively. Four kinds of health conditions are carried out for 10 s, which include Normal, IF, OF and B. Furthermore, the same data preprocessing as case study 1 is executed and the experimental settings are listed in Table 12.

#### 4.4.2. Results Analysis

**Figure 20:**
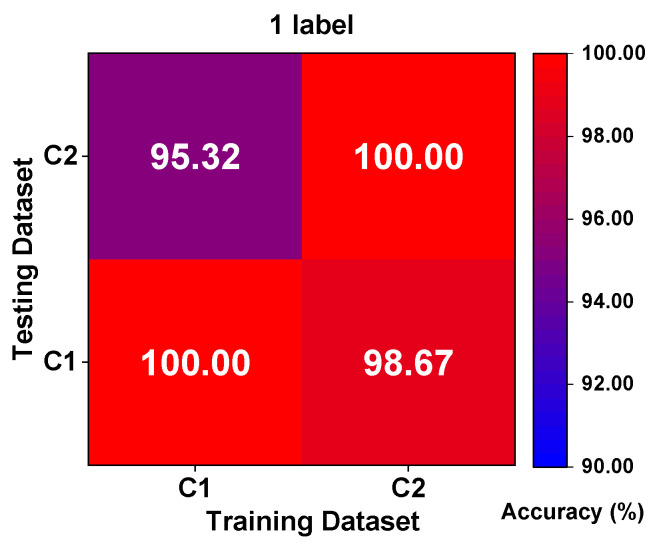
Heatmaps of experimental results with 1 label.

**Figure 21:**
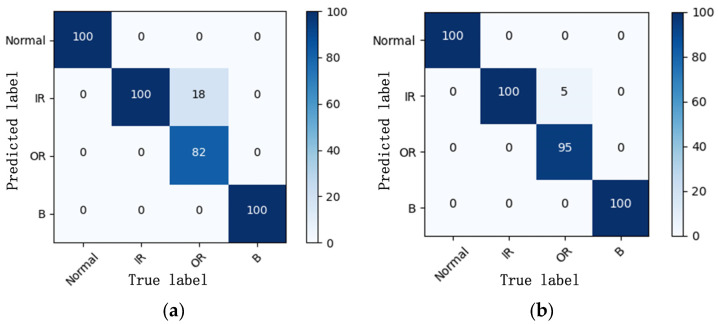
Confusion matrices for cross-working condition task: (**a**) C1–C2; (**b**) C2–C1.

We carried out two typical working conditions including four kinds of identification tasks, respectively, as the most demanding data condition, and the heatmaps of the experimental results for one labeled sample are displayed in Figure 20. It can be seen that the result of the classification task can reach 100% under the same working conditions, which is significantly higher than the experimental results of the CWRU bearing dataset in case study 1. The classification target focusing on fault location is considered a prime reason for this phenomenon, and we can see to some extent that the neglecting of the damage degree reduces the training difficulty for the proposed method. Faced with variable working conditions with one labeled sample, the classification task of the fault location can still reach more than 95%. Observing the confusion matrices in Figure 21, which is the closest to the average accuracy, the experimental results are analyzed separately under two different working conditions. Specifically, only 18 and 5 samples belonging to OR faults are wrongly classified as IR faults, respectively, and none of the faults’ health statuses are misidentified, which can ensure the safe operation of the MSTR long-term reliability test. From the experiments of this case study, we further prove that the proposed Tri-CLAN has extensive applicability and universality. In different application scenarios, it can effectively mine extremely limited labeled data and utilize easily available unlabeled data to achieve fault diagnosis under variable working conditions.

## 5. Conclusions

Given the actual industrial data, it is urgent and necessary to carry out end-to-end intelligent fault diagnosis. This paper innovatively proposes a triplet-guided path-interaction CNN-based ladder network, which realizes the intelligent fault diagnosis with extremely limited labeled samples under variable working conditions. To accommodate the data requirements, the proposed method can be elaborated from the aspects of algorithm structure and feature space. First, to integrate the advantage of the convolution operation and achieve information fusion with fewer parameters, this paper replaces the vanilla LAN backbone with a CNN and constructs a path-interaction semi-supervised architecture with a simplified combination activation function. Furthermore, benefiting from the contribution to the triplet loss with a hard sample mining strategy, the feature distribution shifting problem between variable working conditions is alleviated by learning fault-related representation at the feature level. The public CWRU bearing dataset is utilized to verify the effectiveness of the proposed method, and two experimental datasets are applied to illustrate its extensive applicability. In future research, we will collect data from more engineering application scenarios to verify the proposed algorithm. Furthermore, we will further study the intelligent fault diagnosis method for the purpose of its application to other extreme circumstances in the industry.

## Figures and Tables

**Table 1:** Basic modules.

Module	Network Layer	Kernel Size	Module		Neurons Number
CE-CNN	Conv1d #1	16 × 15	CE-FC		896-512-256
	Conv1d #2	32 × 15	DE-FC		512-896-1024
	Conv1d #3	64 × 5	Predicting	#1	Embedding-1024
DE-CNN	TransConv1d #1	32 × 5		#2	1024-64
	TransConv1d #2	16 × 15		#3	64-fault classes
	TransConv1d #3	1 × 15	Metric learning		~

**Table 2:** Architectures of the methods in case studies.

Methods	CE-CNN	DE-CNN	CE-FC	DE-FC	Predicting	Metric Learning
Tri-CLAN	✓	✓			✓	✓
CLAN	✓	✓			✓	
Tri-CNN	✓				✓	✓
CNN	✓				✓	
Tri-LAN			✓	✓	✓	✓
Vanilla LAN			✓	✓	✓	

**Table 3:** Parameter setup for experiments.

Parameters	Values
Learning rate	0.001
Training epochs	100
Batch size of labeled data	fault classes
Batch size of unlabeled data	200
Gaussian noise ε	(0,1)

**Table 4:** Working conditions of CWRU.

Working Condition	Motor Load (hp)	Motor Speed (rpm)
C1	0	1797
C2	1	1772
C3	2	1750
C4	3	1730

**Table 5:** Labels of fault location with different damage degree.

Fault Location	Fault Diameter (Inch)	Fault Labels
None (Normal)	0	0
Inner Raceway (IR)	0.07	1
	0.14	2
	0.21	3
Outer Raceway (OR)	0.07	4
	0.14	5
	0.21	6
Ball (B)	0.07	7
	0.14	8
	0.21	9

**Table 6:** Experiment 1: settings of datasets under C1.

Name	Training Samples (Labeled/Unlabeled)	Testing Samples
A_1_	5/100	100
B_1_	2/100	100
C_1_	1/100	100

**Table 7:** Experiment 2: settings of datasets under variable working conditions.

Name	Training Samples (Labeled/Unlabeled)				Testing Samples			
	C1	C2	C3	C4	C1	C2	C3	C4
A_2_	1/100	0	0	0	100	100	100	100
	2/100							
	5/100							
B_2_	0	1/100	0	0				
		2/100						
		5/100						
C_2_	0	0	1/100					
			2/100					
			5/100					
D_2_	0	0	0	1/100				
				2/100				
				5/100				

**Table 8:** Comparisons with state-of-the-art methods for experiment 1 on CWRU.

References	Fault Location	Damage Degree	Training Samples (Labeled/Unlabeled)	Accuracy (%)
[42]	✓	✓	50/950	98.40
[43]	✓	-	10/-	90.93
[44]	✓	-	900/-	88.54
[19]	✓	✓	300/12,900	87.63
This work	✓	✓	50/1000	99.98
			20/1000	99.43
			10/1000	92.45

**Table 9:** Working conditions of the motor fault dataset.

Working Condition	Setting Speed (rpm)	Actual Speed (rpm)	Load (N·m)
C1	1750	1722	33
C2	1500	1490	17
C3	1750	1740	17
C4	900	875	33

**Table 10:** Labels of motor fault location.

Fault Location	Fault Labels
Normal	0
Rotor unbalanced motor (RUM)	1
Bending rotor motor (BRM)	2
Faulty bearing motor (FBM)	3
Broken bar motor (BBM)	4
Stator winding fault motor (WFM)	5
Single phase fault motor (SPM)	6

**Table 11:** Experimental settings of motor datasets under variable working conditions.

Name	Training Samples (Labeled/Unlabeled)				Testing Samples			
	C1	C2	C3	C4	C1	C2	C3	C4
A	1/100	0	0	0	100	100	100	100
B	0	1/100	0	0				
C	0	0	1/100	0				
D	0	0	0	1/100				

**Table 12:** Experimental settings of MSTR under variable working conditions.

Name	Training Samples (Labeled/Unlabeled)		Testing Samples	
	C1	C2	C1	C2
A	1/100	0	100	100
B	0	1/100		

## Data Availability

Data available on request due to restrictions.
